# How public health authorities can use pathogen genomics in health protection practice: a consensus-building Delphi study conducted in the United Kingdom

**DOI:** 10.1099/mgen.0.000912

**Published:** 2023-02-06

**Authors:** Nicholas Killough, Lynsey Patterson, The COVID-19 Genomics UK (COG-UK) consortium†, Sharon J. Peacock, Declan T. Bradley

**Affiliations:** ^1^​ Public Health Agency, Belfast, UK; ^2^​ Centre for Public Health, Queen’s University Belfast, Belfast, UK; ^3^​ Department of Medicine, University of Cambridge, Cambridge, UK

**Keywords:** COVID-19, Delphi Study, Health Protection, Pathogen genomics, SARS-CoV-2, Sequencing

## Abstract

Pathogen sequencing guided understanding of SARS-CoV-2 evolution during the COVID-19 pandemic. Many health systems developed pathogen genomics services to monitor SARS-CoV-2. There are no agreed guidelines about how pathogen genomic information should be used in public health practice. We undertook a modified Delphi study in three rounds to develop expert consensus statements about how genomic information should be used. Our aim was to inform health protection policy, planning and practice. Participants were from organisations that produced or used pathogen genomics information in the United Kingdom. The first round posed questions derived from a rapid literature review. Responses informed statements for the subsequent rounds. Consensus was accepted when 70 % or more of the responses were strongly agree/agree, or 70 % were disagree/strongly disagree on the five-point Likert scale. Consensus was achieved in 26 (96 %) of 27 statements. We grouped the statements into six categories: monitoring the emergence of new variants; understanding the epidemiological context of genomic data; using genomic data in outbreak risk assessment and risk management; prioritising the use of limited sequencing capacity; sequencing service performance; and sequencing service capability. The expert consensus statements will help guide public health authorities and policymakers to integrate pathogen genomics in health protection practice.

## Data Summary

The authors confirm all supporting data, code and protocols have been provided within the article or through supplementary data files. The survey responses are included in Supplementary Materials.

**Impact Statement**

Our study will inform public health authorities, commissioners and healthcare providers about how pathogen genomics can be used in practice. The consensus statements developed through the Delphi process provide a framework for organisations to assess their own capability, and to inform the future development and integration of pathogen genomics in practice.

## Introduction

The specialist area of pathogen genomics has assumed increasing prominence during the COVID-19 pandemic, and sequencing of SARS-CoV-2 has been performed on a much greater scale than for any previous pathogen [1]. The COVID-19 Genomics UK (COG-UK) Consortium was established rapidly in response to the COVID-19 pandemic. COG-UK leveraged expertise, people and equipment from diverse organisations as part of a single national sequencing programme. Its role in research, surveillance and policy-making has been central to the delivery of a pathogen genomics service and capability for the United Kingdom (UK). The rapid development of consortium tools allowed for harmonised analysis and reporting, and sharing of data and intelligence in the UK and internationally [2]. Sequence data and lineages were made publicly available through GISAID and the COG-UK website [3–5].

Sequencing has informed our understanding of the evolution of the SARS-CoV-2 virus. However, there is limited practical guidance for health protection teams in public health authorities about how pathogen genomics data should be used in practice; for example, in terms of risk assessment and risk management of cases, clusters and outbreaks. Policy-makers and service commissioners also need information to support decisions about how to fund pathogen genomics services.

The Delphi consensus-building research method aims to develop expert consensus statements [6]. This method is appropriate where the goal is to allow a group of individuals to take part in ‘structured communication’ about a complex issue [7], such as developing clinical guidelines. For example, the Delphi method was used in the context of COVID-19 to create expert clinical practice statements on the management of respiratory failure [8]. As part of the Public Health Agency’s COG-UK-funded Public health Risk Assessment using Genomic Methods And Tools In Context (PRAGMATIC) Study, we planned and undertook a Delphi study. Our study aimed to develop a set of expert consensus statements that could guide public health authorities about how they should use pathogen sequencing to support health protection risk assessment and outbreak management.

## Methods

### Study design

To identify baseline information to inform the development of our study, in September 2021 we undertook a rapid review of published reports on the use of pathogen sequencing in public health practice. We searched PubMed, using the following keywords and phrases: ‘public health genomic sequencing’, ‘whole genome sequencing in public health’, and ‘sequencing and public health action’. We restricted the results to those published in English between January 2018 and August 2021. We chose not to undertake a formal systematic literature review due to our assessment that it would not be feasible to conduct a systematic review, Delphi study and to implement the findings in our practice during the 6 month funded timeline of our project. We therefore chose to use a modified Delphi method in which we asked the expert respondents to provide free-text responses in the first round of the study to inform the development of the second-round statements [9, 10].

The major themes identified in our rapid review highlighted the importance of specific contexts, such as the use of sequencing in identifying variants, the investigation of the effects of transmission, immune escape and severity of disease. It was reported that sequencing played a key role in outbreak investigation within healthcare and residential care settings. The literature described methods of interpretation and dissemination of sequencing results to key stakeholders. These themes formed the basis of our round one questions.

### Participant recruitment

Forty-four people were invited by email to participate in the study. The invitees were from institutions associated with the production or use of pathogen genomics information in the UK, including COVID-19 Genomics UK Consortium (COG-UK) members, the four UK public health agencies, healthcare providers and academic researchers. Participants were initially identified through our professional networks, and onwards through a snowballing approach. The participants were from a range of professional backgrounds, including scientists, clinicians, policymakers, researchers, and public health practitioners. Thirty-three potential participants were identified through their roles in the UK’s four public health agencies (in health protection or pathogen genomics services), eight were primarily affiliated to academic organisations, and three were affiliated to National Health Service or Health and Social Care Trust laboratories. Nineteen of 33 invitees from public health agencies agreed to participate (58 %), five of eight primarily academic invitees agreed (63 %), and one of three Trust laboratory invitees agreed (33 %). The research team did not participate in the study as respondents. We chose not to collect demographic characteristics of respondents as due to the small number of participants we assessed that it would be possible to identify individuals by the unique combinations of characteristics, which would have undermined the anonymous nature of the study. We asked whether the participant was a producer or user (or both) of pathogen sequence data. If participants missed a round, they were eligible to take part in a later round, as this is thought to reduce the risk of false consensus and to better reflect the views of the invited participants [11].

### Delphi process

We invited the participants to take part in three rounds of an online survey, which took place between 5 and 12 January 2022, 1 and 10 March 2022, and 11 March and 22 March 2022. One reminder was sent to all participants during each round. The first round comprised twenty questions with free-text answer fields (Table S1, available in the online version of this article). From this, we developed the second-round statements, which required responses on the Likert Scale from 1 (strongly agree) to 5 (strongly disagree) (Supplementary Materials). Consensus was accepted when 70 % or more of the responses were either strongly agree/agree, or alternatively, 70 % or more of responses were disagree/strongly disagree, on the five-point Likert scale [12–15]. When consensus was not achieved, statements were revised to improve the clarity or specificity, and were carried through into a subsequent round. The Delphi exercise ended after round three if statements did not reach consensus.

### Data analysis and reporting

The expert consensus statements were tabulated and presented alongside explanatory notes informed by the authors’ summary of the participants’ free-text responses. We followed the Conducting and Reporting of Delphi Studies (CREDES) guidance [6].

## Results

### Study population

**Fig. 1.**
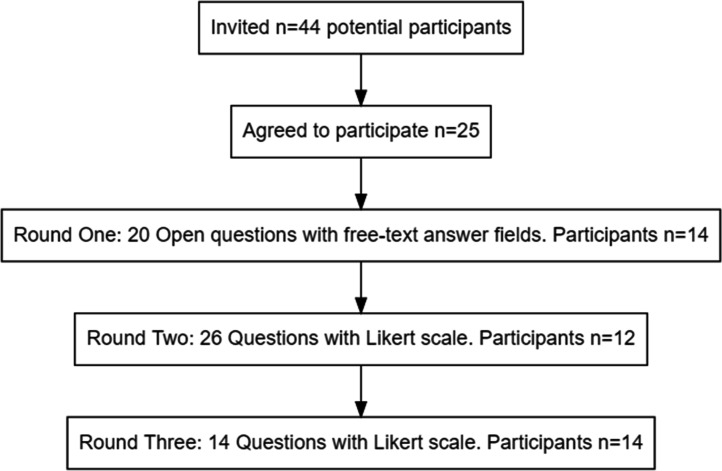
Study flowchart

Twenty-five participants (57 %) responded to the initial invite and agreed to participate in the study. Twelve to fourteen of those responded to each survey round. They were a mix of people who produced and used pathogen genomic data (Table 1; Fig. 1).

**Table 1.** Study population

	Round one *N*=14	Round two *N*=12	Round three *N*=14
Producer only	2 (14 %)	1 (8 %)	1 (7 %)
User	8 (57 %)	7 (59 %)	9 (64 %)
Both user and producer	4 (29 %)	4 (33 %)	4 (29 %)

### Delphi round one completion

Twenty questions with a free-text answer field were asked in round one. Fourteen of 25 participants responded. The questions were answered with a range of completeness. Thirteen out of the 20 (65 %) questions were answered by more than ten participants while seven of the 20 (35 %) questions were answered by fewer than eight participants (Table 2). The full responses are in Supplementary Materials.

**Table 2.** Rounds two and three completion

Round	Participants	Questions	Consensus	Consensus not achieved
2	12/25 (48 %)	26	18 (69 %)	8 (31 %)
3	14/25 (56 %)	9	8 (89 %)	1 (11 %)

### Delphi rounds two and three completion

In round two, a consensus was reached for 18 (69 %) of the 26 statements presented (Summarised in Table 3, full results in Supplementary Materials). In round three, one question did not reach consensus. We concluded that consensus would not be achieved following a further round of questioning for this statement (Table 3). The Delphi study was concluded after round three.

**Table 3.** Summary quantitative results of rounds two and three

Question	Strongly agree / Agree No. (%)	Neither agree nor disagree No. (%)	Strongly disagree / Disagree No. (%)
**Round 2**			
Q2. Public health authorities should use pathogen genomic sequencing to detect the emergence of new variants.	12 (100.0)	0 (0)	0 (0)
Q3. Public health authorities should use pathogen genomic sequencing as a surveillance tool to monitor geographical spread of variants over time.	12 (100.0)	0 (0)	0 (0)
Q4. Public health authorities and healthcare providers should use pathogen genomic sequencing in outbreak investigations in health care settings to rule in or rule out transmission events.	10 (83.3)	2 (16.7)	0.0
Q5. Public health authorities should use pathogen genomic data to inform estimates of epidemic growth.	10 (83.3)	2 (16.7)	0.0
Q6. Public health authorities should use pathogen genomic data to inform estimates of clinical severity of the disease.	11 (91.7)	1 (8.3)	0.0
Q7. Public health authorities should use pathogen genomic data to detect changes in risk of infection in specific settings e.g., schools, care homes etc.	9 (75.0)	3 (25.0)	0.0
Q8a. Pathogen genomic information should inform evaluation of the effect of: non-pharmaceutical interventions.	6 (50.0)	4 (33.3)	2 (16.7)
Q8b. Pathogen genomic information should inform evaluation of the effect of: vaccines.	11 (91.7)	1 (8.3)	0.0
Q8c. Pathogen genomic information should inform evaluation of the effect of: pharmaceutical therapeutic treatments.	10 (83.3)	2 (16.7)	0.0
Q9. Pathogen genomic information should be linked to contextual epidemiological information to facilitate risk assessment.	11 (91.7)	1 (8.3)	0.0
Q10. Public health authorities should prioritise sequencing from outbreaks where it appears there is greater than expected disease severity.	11 (91.7)	1 (8.33)	0.0
Q11. Public health authorities should sequence enough randomly selected samples to enable unbiased surveillance.	12 (100.0)	0.0	0.0
Q12. Public health authorities should prioritise sequencing of samples from vaccinated people.	8 (66.7)	3 (25.0)	1 (8.3)
Q13. Public health authorities should prioritise sequencing of samples from people who have had multiple episodes of infection.	10 (83.3)	1 (8.3)	1 (8.3)
Q14. If it is not possible to sequence all samples, public health authorities should direct sequencing capacity towards vulnerable populations.	8 (66.7)	1 (8.3)	3 (25.0)
Q15. If it is not possible to sequence all samples, public health authorities should direct sequencing capacity towards outbreak investigation.	7 (58.3)	3 (25.0)	2 (16.7)
Q16. If it is not possible to sequence all samples, public health authorities should direct sequencing capacity towards populations in whom new variants might be present e.g., international travellers and immunocompromised people.	12 (100.0)	0.0	0.0
Q17. Public health authorities should use pathogen genomic data to de-escalate potential outbreaks that were identified through epidemiological links.	7 (58.3)	4 (33.3)	1 (8.3)
Q18. Timeliness of genomic sequencing results is important to allow results to be acted upon by public health authorities.	12 (100.0)	0.0	0.0
Q19. Clinical teams need timely access to sequence results to inform treatment and infection control decisions.	11 (91.7)	0.0	1 (8.3)
Q20. A minimum of ten percent of all positive COVID-19 samples should be sequenced.	7 (58.3)	5 (41.7)	0.00
Q21. Public health authorities should use tools such as rapid genotyping for surveillance of lineages.	9 (75.0)	3 (25.0)	0.00
Q22. Public health authorities should analyse sequence and/or single nucleotide polymorphism data as part of investigation of outbreaks.	8 (66.7)	3 (25.0)	1 (8.3)
Q23. Analysts should exclude sequences below a defined sequence coverage from analysis when investigating transmission events.	4 (33.3)	6 (50.0)	2 (16.7)
Q24. Public health authorities should use bioinformatics tools to elicit unsuspected transmission events.	10 (83.3)	2 (16.7)	0.0
Q25. Public health authorities should ensure training is provided for health protection, infection control and clinical teams on the interpretation of sequencing results.	12 (100.0)	0.0	0.0
**Round 3**			
Q2. If it is not possible to sequence all samples, public health authorities should direct sequencing capacity towards people who are at greater risk of adverse outcomes from infection.	10 (71.4)	0.00	4 (28.6)
Q3a. If it is not possible to sequence all samples, public health authorities should direct sequencing capacity towards outbreak investigations: In closed settings, such as care homes and hospitals.	13 (92.9)	1 (7.1)	0.0
Q3b. If it is not possible to sequence all samples, public health authorities should direct sequencing capacity towards outbreak investigations: In community outbreaks, such as at public events or functions.	10 (71.4)	4 (28.6)	0.0
Q4a. Public health authorities should use pathogen genomic data to de-escalate potential outbreaks that were identified through epidemiological links: In closed settings, such as care homes and hospitals.	13 (92.9)	1 (7.1)	0.0
Q4b. Public health authorities should use pathogen genomic data to de-escalate potential outbreaks that were identified through epidemiological links: in community outbreaks, such as at public events or functions.	10 (71.4)	3 (21.5)	1 (7.1)
Q5. Pathogen genomic information should inform evaluation of the effect of non-pharmaceutical interventions.	10 (71.4)	4 (28.6)	0.0
Q6. The proportion of samples sequenced should reflect the epidemiological context.	12 (85.7)	2 (14.3)	0.0
Q7. Public health authorities should analyse sequencing data in more detail than lineage as part of the investigation of outbreaks.	13 (92.9)	0.0	1 (7.1)
Q8. Analysts should only include sequences above defined sequence coverage depth when investigating transmission events. (This is an overall quality control value provided for all sequencing results).	8 (57.1)	4 (28.6)	2 (14.3)
**Questions that failed to reach consensus**			
Round 2: Analysts should exclude sequences below a defined sequence coverage from analysis when investigating transmission events.	4 (33.3)	6 (50.0)	2 (16.7)
Round 3: Analysts should only include sequences above defined sequence coverage depth when investigating transmission events. (This is an overall quality control value provided for all sequencing results).	8 (57.1)	4 (28.6)	2 (14.3)

### Statements that achieved consensus

The statements that achieved consensus, with accompanying explanatory notes, are presented in Table 4.

**Table 4.** A summary of consensus statements and the rationale provided by the expert panel

Statement no.	Expert statement	Explanation
**Monitoring the emergence of new variants**		
1.	Public health authorities should use pathogen genomic sequencing to detect the emergence of new variants.	Participants and published literature highlighted the importance of sequencing for detecting the evolution and emergence of new variants of pathogens [19, 20].
2.	Public health authorities should use pathogen genomic sequencing as a surveillance tool to monitor geographical spread of variants over time.	Strong support was given to the use of sequencing as a tool to monitor the geographical spread of variants after their initial introduction to a population [21].
3.	Public health authorities should sequence enough randomly selected samples to enable unbiased surveillance.	Participants highlighted the importance of unbiased surveillance to ensuring that variants are detected as part of routine surveillance. It was emphasised that this is essential in detecting changes in the prevalence of variants and ensuring the variants believed to be extinct are no longer detected [22].
**Understanding the epidemiological context of genomic sequence data**		
4.	The proportion of samples sequenced should reflect the epidemiological context.	The absolute number and relative proportion of positive samples sequenced will affect the confidence that can be placed on the results for risk assessment and decision-making. ECDC has published guidelines that pathogen genomics surveillance systems should sequence a minimum of 10 % of positive samples and they discuss the importance of linking this information to local public health systems [23–25].
5.	Pathogen genomic information should be linked to contextual epidemiological information to facilitate risk assessment.	Sequencing may be used as a tool to support or discount potential transmission events in those identified as having epidemiological links. Epidemiological, demographic and clinical data can supplement genomic data as part of analyses of severity and vaccine escape. When the aim is to interrupt community transmission, ongoing contact tracing of linked clusters may help prioritise the sequencing of community isolates [26].
**Use of genomic data in outbreak risk assessment and risk management**		
6.	Public health authorities and healthcare providers should use pathogen genomic sequencing in outbreak investigations in health care settings to rule in or rule out transmission events.	Much published work has focused on the potential of genomics to identify unrecognised connections between cases, but less attention has been given to the importance of ruling out transmission events, which can effectively disprove transmission between individuals and thus de-escalate a situation. This has recently been addressed with the development of tools like the HOCI Sequence Reporting Tool (SRT) [27]. Tools should contextualise the probability of transmission. It was suggested by participants that maximum likelihood phylogenetic trees should be used, at least at a local level and that further detail should be provided with time-scaled trees, such as those that can be created with IQTREE-2 or BEAST 2 [28, 29].
7.	Public health authorities should use pathogen genomic data to de-escalate potential outbreaks that were identified through epidemiological links in closed settings, such as care homes and hospitals.	Sequence results have demonstrated the ability to de-escalate outbreaks by excluding nosocomial transmission when multiple external infection introductions have been identified [27, 30]. This may facilitate the reallocation of resources away from potential incidents that were shown to be unrelated cases.
8.	Public health authorities should use pathogen genomic data to de-escalate potential outbreaks that were identified through epidemiological links in community outbreaks, such as at public events or functions.	When assessing outbreaks in a community setting identifying the source of infection can be very difficult. Pathogen sequencing has helped demonstrate transmission that can often occur in large community settings such as public events [31].
9.	Public health authorities should use pathogen genomic data to inform estimates of epidemic growth.	Pathogen genomics is essential in assessing the spread of emerging variants. It has played a key role in the understanding of the effects of mutations on the growth advantage of new variants [32].
10.	Public health authorities should use pathogen genomic data to inform estimates of clinical severity of the disease.	During the COVID-19 pandemic, the transition between variants has been accompanied by differences in clinical severity and vaccine effectiveness. Linked pathogen genomic data is important for measuring these factors [33].
11.	Public health authorities should use pathogen genomic data to detect changes in risk of infection in specific settings e.g., schools, care homes etc.	Understanding the relative attack rates associated with different lineages or variants in specific setting may help public health authorities assess and manage risk in those settings [27].
12.	Pathogen genomic information should inform evaluation of the effect of vaccines and pharmaceutical therapeutic treatments.	In the context of COVID-19, sequence data have supported detection of reductions in vaccine effectiveness associated with the emergence of new variants [34–36] and informed booster vaccine schedules [37]. The use of novel COVID-19 therapies has been guided by knowledge of the variant at an individual and population level [38].
13.	Pathogen genomic information should inform evaluation of the effect of non-pharmaceutical interventions.	The effectiveness of individual actions (such as social distancing and personal protective equipment) and societal restrictions (such as the mandated closure of places of business and education) were affected by SARS-CoV-2 variants, with respect to both the transmissibility and severity. Timely genomic data was important for managing these situations [39–42].
**Prioritising the use of limited sequencing capacity**		
14.	Public health authorities should prioritise sequencing from outbreaks where it appears there is greater than expected disease severity.	Outbreaks with a significantly greater than expected case hospitalisation ratio or case fatality ratio could be an early sign of a variant with greater inherent severity or immune escape, and should result in sequencing to assess this risk [43, 44].
15.	Public health authorities should prioritise sequencing of samples from vaccinated people.	New variants of SARS-CoV-2 that became dominant after the introduction of the vaccination programme were associated with progressively reduced vaccine effectiveness, possibly due to the selection pressure in the context of high prevalence and high vaccination coverage. Identifying changes in vaccine effectiveness provides opportunities for changes to vaccines or to introduce additional doses to manage the impact of such antigenic changes [43].
16.	Public health authorities should prioritise sequencing of samples from people who have had multiple episodes of infection.	Reinfection may indicate immune escape due to the emergence of new variants, and should be monitored using integrated genomic and epidemiological data [45, 46].
17.	If it is not possible to sequence all samples, public health authorities should direct sequencing capacity towards outbreak investigations in closed settings, such as care homes and hospitals.	Participants emphasised the importance of using genomic information to determine the need for implementing additional protective measures in closed settings during outbreaks [47]. Application of stricter measures may be required in an outbreak of a high-risk variant in a closed setting such as in a care home whereas, an outbreak of the dominant lineage may not warrant the same additional measures. In a hospital however, we see a far more open setting with introductions possible from the community at a more regular interval. In this case we see a greater proportion of spread to wards from outpatient clinics, staff as well as between wards. In this scenario, a more consistent and higher coverage of sequencing may be of use to identify problematic areas and implement IPC measures earlier e.g., improved ventilation, reinforcing mask wearing rules, hand hygiene, etc [48].
18.	If it is not possible to sequence all samples, public health authorities should direct sequencing capacity towards populations in whom new variants might be present e.g., international travellers.	When a new variant emerges in one place, there will be a lead time before it reaches other locations. International borders present an opportunity to detect new variants by effectively sampling people who have recent exposure in other regions. Early awareness of new variants can inform the response to their arrival in a timely way [49, 50].
19.	If it is not possible to sequence all samples, public health authorities should direct sequencing capacity towards populations in whom new variants might be present e.g., immunocompromised people.	The risk of the evolution of new variants may be increased in individuals who are immunocompromised, due to the selection pressure arising from ineffective immune response and consequent longer infection duration. Participants believed that these individuals should be prioritised due to the opportunity to detect new variants in the first person in whom a variant arose and the opportunity to respond to this situation [51]. People who receive treatment with antiviral or antibody therapies, which may create a selection pressure towards resistance, may also be prioritised.
20.	If it is not possible to sequence all samples, public health authorities should direct sequencing capacity towards people who are at greater risk of adverse outcomes from infection.	When there is diversity of variants in circulation, information about which variants are responsible for infection in demographic groups that experience severe outcomes may help inform public health risk assessment and management, and wider policy choices aimed at reducing harm [52].
21.	If it is not possible to sequence all samples, public health authorities should direct sequencing capacity towards outbreak investigations in community outbreaks, such as at public events or functions.	The ability to identify the transmission pathways may highlight opportunities for future prevention, or alternatively, sequencing may disprove transmission and change the risk assessment of behaviours or events [53].
**Sequencing service performance**		
22.	Timeliness of genomic sequencing results is important to allow results to be acted upon by public health authorities.	Timely results enable the assessment of the growth rates of new lineages and can inform advice about mitigation. Rapid genotyping can be a useful adjunct to sequencing for the monitoring of new variants but requires a high level of specificity [54]. Timely genomic results can help inform public health risk management in outbreaks [44].
23.	Clinical teams need timely access to sequence results to inform treatment and infection control decisions.	In the early stages of an outbreak of a new variant, sequencing results have been used to inform treatment decisions, which is therefore time-sensitive [55]. Identifying the introduction and transmission of a lineage within a hospital, for example, can reveal breaks in infection control and inform infection prevention and control measures [40, 47].
**Sequencing service capability**		
24.	Public health authorities should ensure training is provided for health protection, infection control and clinical teams on the interpretation of sequencing results.	Interpretation and analysis of pathogen genomics results require a range of multidisciplinary skills. Participants highlighted: data processing and epidemiological analysis; outbreak investigation and management; infection prevention and control methods and factors associated with transmission; knowledge of pathogen biology and evolution; understanding of genomics terms, such as SNP-distance, time to most recent common ancestor (TMRCA), cluster and lineage; ability to interpret phylogenetic trees; understanding of the process and limitations of sequencing; understanding of what sequencing data represents and how it is generated; understanding of the process for selecting samples and the proportion of samples being sequenced at a given; understanding the limitations of SARS-CoV-2 sequencing; the ability to integrate genomic cluster data with epidemiological data at scale; knowledge of infection prevention and control (IPC) practice; understanding of the IPC implications for genomically-linked and genomically-refuted clusters; understanding the limitations of epidemiological information; and understanding the application of genomics in investigating vaccine effectiveness and immune escape.
25.	Public health authorities should use tools such as rapid genotyping for surveillance of lineages.	Genotyping methods have supported epidemiological surveillance and public health response, but preparation of relevant assays requires prior knowledge of the haplotypic relationship between specific SNPs and a lineage of interest. It is a compromise of increased speed (and reduced cost) compared to the more detailed genomic information provided by sequencing [53]. Genotyping provides less resolution and cannot be used to create transmission trees, for example.
26.	Public health authorities should use bioinformatics tools to elicit unsuspected transmission events.	With the increasing availability and the development of bioinformatics tools like CIVET and outbreaker2 the ability to investigate unsuspected transmission events has become more comprehensive. These tools allow investigators to look beyond the local level of outbreaks and build connections not previously identified [56, 57].
27.	Public health authorities should analyse sequencing data in more detail than lineage as part of the investigation of outbreaks.	Genomic information at the level of genomic lineage can indicate where transmission has not occurred. It is common, however, when a lineage is dominant, for most infections to be of the same lineage. More detailed analysis can provide further information in outbreak scenarios of the same lineage [57]. Tools that can infer the probability of transmission, like those developed in the HOCI study, should be used to investigate outbreaks [27]. Where possible maximum likelihood phylogenetic trees should be used.

### Statements that did not reach consensus

Statements that did not reach consensus after the third Delphi round are presented in Table 5.

**Table 5.** A summary of the consensus statement that did not reach consensus and the rationale provided by the study panel.

Statement no.	Expert statement	Explanation
**Use of genomic data in transmission investigations**		
1.	Analysts should only include sequences above defined sequence coverage depth when investigating transmission events. (This is an overall quality control value provided for all sequencing results).	In the first iteration of this statement, responses demonstrated a need for greater clarity about the implications of sequencing coverage for interpretation of lineage calls. The statement was twice revised for precision and clarity, and did not reach consensus.

### Pathogen genomics bioinformatics tools described in expert responses

During the completion of the first round of free-text answers, a series of bioinformatics tools were suggested for analysis of sequence data (Table 6).

**Table 6.** Pathogen genomics bioinformatics tools described in expert responses

Tool	Synopsis
A2B COVID	‘A2B-COVID: A Tool for Rapidly Evaluating Potential SARS-CoV-2 Transmission Events’ [58].
BEAST 2	‘BEAST two is a cross-platform programme for Bayesian phylogenetic analysis of molecular sequences. It estimates rooted, time-measured phylogenies using strict or relaxed molecular clock models. It can be used as a method of reconstructing phylogenies but is also a framework for testing evolutionary hypotheses without conditioning on a single tree topology’ [29].
Civet	‘Cluster Investigation and Virus Epidemiology Tool civet is a tool developed with 'real-time' genomics in mind. Using a background phylogeny, such as the large phylogeny available through the COG-UK infrastructure on CLIMB, civet will generate a report for a set of sequences of interest i.e., an outbreak investigation’ [56].
HOCI Sequence Reporting Tool (SRT)	‘The COG-UK Consortium Hospital-Onset COVID-19 Infections (COG-UK HOCI) study aims to evaluate whether the use of rapid whole-genome sequencing of SARS-CoV-2, supported by a novel probabilistic reporting methodology, can inform infection prevention and control (IPC) practice within NHS hospital settings’ [27].
IQTREE-2	‘IQTREE-2 is a fast and effective stochastic algorithm to infer phylogenetic trees by maximum likelihood. IQ-TREE compares favourably to RAxML and PhyML in terms of likelihoods with similar computing time’ [28].
Transcluster	‘Transcluster is an R package for inferring and viewing transmission clusters from sequence alignments and sample dates’ [59].

## Discussion

The COVID-19 pandemic highlighted the need for guidance on the use of pathogen sequencing for policymakers and practitioners. Our study provides expert consensus statements to support investigators and researchers to develop services. The statements should inform the development of systems and processes for delivering and monitoring pathogen genomics services, and inform practical operational use of pathogen genomic information by public health teams. The themes and statements may be used as a self-assessment tool by public health agencies in the development of their capabilities. Delphi studies can also reveal points of professional controversy or disagreement, i.e. dissensus. Our study only resulted in one statement about which there was disagreement, which was about the potential use of sequence data in outbreak investigation where the data did not pass (unspecified) thresholds of sequence depth or quality. This may reflect differing technical knowledge of participants, or true disagreement about the technical limits at which data can be interpreted with validity. The qualitative responses from participants in round one revealed information about bioinformatics tools for practical use of pathogen genomics data in public health practice, which may be useful for teams that are developing their capabilities.

Pathogen genomic sequencing is now used as part of the public health management of infection with *Mycobacterium tuberculosis *, *Campylobacter* spp., *Staphylococcus aureus *, *Escherichia coli *, *Shigella* spp., *Listeria* spp. and *Salmonella* spp. [16, 17]. The use of pathogen genomics in public health practice may inform direct clinical care and the effectiveness of outbreak management [18]. The COVID-19 pandemic highlighted the need for public health agencies to have multidisciplinary pathogen genomics capability, supported by effective data infrastructure and processes. Timely genomic results integrated with epidemiological data can support public health teams to assess and manage the risk of outbreaks and wider epidemics in real time. HDR-UK and CLIMB-COVID have supported the integration of COG-UK genomic data with pseudonymised data for research purposes, and genomic data are available through the trusted research environments of the UK nations.

The consensus statements reached in our study reflect a specific point in the management of the COVID-19 pandemic, and the capability, capacity and maturity of UK pathogen genomics services. Though there are generalisable lessons from this work beyond COVID-19 and into the future, as the policy, testing, practice, methods and funding situations evolve over time, the findings may become less relevant. The Delphi method is readily usable in a new situation or context, should a need to update recommendations develop.

### Strengths and limitations

As the participants were individuals who created and used pathogen genomics data during the COVID-19 pandemic the findings highlight the real-world, practical issues that they faced in the implementation and application of a pathogen genomics service.

The number of participants was relatively low, and we limited invitations to people who were working within the UK public health and genomics system. The timing of our study coincided with the emergence of the Omicron variant, which doubtless competed for the attention of our invited potential participants. The short windows for completion of the study rounds may have limited the response rate. The Delphi study method inherently incorporates some subjectivity from the participants and the researchers, which we have aimed to mitigate through our transparent adherence to a conduct and reporting standard, and open data sharing with our report [7]. There is no standard method for confirming consensus in Delphi studies [14]. The 70 % threshold for agreement that we used reflects a *degree* of consensus, about which there is no objective gold standard to measure against. Alternative rules or thresholds could have resulted in variation in our results.

Our study focused on gaining expert consensus from individuals who work in pathogen services, or who directly use the information that those services produce. As such, the study did not have patient, public or service user participants. We did not have any patient or public members of our research team. We believe that it is important that future work should have patient and public representation in their design and conduct.

## Conclusion

Pathogen genomics capability has been greatly enhanced by the investment and focus that resulted from the COVID-19 pandemic. The expert consensus statements from the PRAGMATIC Delphi study participants will help public health authorities and policymakers plan for the longer-term applications and integration of pathogen genomics in health protection practice.

## Supplementary Data

Supplementary material 1

Supplementary material 2

## References

[1] (2022). UK completes over 2 million SARS-CoV-2 whole genome sequences - GOV.UK (Internet). https://www.gov.uk/government/news/uk-completes-over-2-million-sars-cov-2-whole-genome-sequences.

[2] (2022). History of COG-UK | COVID-19 Genomics UK Consortium (Internet). https://www.cogconsortium.uk/about/about-us/history-of-cog-uk/.

[3] (2022). Public Data & Analysis | COVID-19 Genomics UK Consortium (Internet). https://www.cogconsortium.uk/priority-areas/data-linkage-analysis/public-data-analysis/.

[4] Wright DW, Harvey WT, Hughes J, Cox M, Peacock TP (2022). Tracking SARS-CoV-2 mutations and variants through the COG-UK-mutation explorer. Virus Evol.

[5] (2022). GISAID - Initiative (Internet). https://www.gisaid.org/.

[6] Jünger S, Payne SA, Brine J, Radbruch L, Brearley SG (2017). Guidance on Conducting and REporting DElphi Studies (CREDES) in palliative care: recommendations based on a methodological systematic review. Palliat Med.

[7] Linstone HA, Turoff M, Helmer O The Delphi Method Techniques and Applications.

[8] Nasa P, Azoulay E, Khanna AK, Jain R, Gupta S (2021). Expert consensus statements for the management of COVID-19-related acute respiratory failure using a Delphi method. Crit Care.

[9] Jones J, Hunter D (1995). Consensus methods for medical and health services research. BMJ.

[10] Dalkey N (1969). An experimental study of group opinion: the delphi method. Futures.

[11] Boel A, Navarro-Compán V, Landewé R, van der Heijde D (2021). Two different invitation approaches for consecutive rounds of a Delphi survey led to comparable final outcome. J Clin Epidemiol.

[12] Slade SC, Dionne CE, Underwood M, Buchbinder R (2014). Standardised method for reporting exercise programmes: protocol for a modified Delphi study. BMJ Open.

[13] Henderson EJ, Rubin GP (2012). Development of a community-based model for respiratory care services. BMC Health Serv Res.

[14] Diamond IR, Grant RC, Feldman BM, Pencharz PB, Ling SC (2014). Defining consensus: a systematic review recommends methodologic criteria for reporting of Delphi studies. J Clin Epidemiol.

[15] Vogel C, Zwolinsky S, Griffiths C, Hobbs M, Henderson E (2019). A Delphi study to build consensus on the definition and use of big data in obesity research. Int J Obes.

[16] UKHSA TB action plan for England. 2021 (cited 2022 Jun 15). https://assets.publishing.service.gov.uk/government/uploads/system/uploads/attachment_data/file/998158/TB_Action_Plan_2021_to_2026.pdf.

[17] Grant K, Jenkins C, Arnold C, Green J, Zambon M (2022). Implementing pathogen genomics implementing pathogen genomics: a case study. https://assets.publishing.service.gov.uk/government/uploads/system/uploads/attachment_data/file/731057/implementing_pathogen_genomics_a_case_study.pdf.

[18] Luheshi L, Raza S, Moorthie S, Hall A, Blackburn L Pathogen genomics into practice. 2015 (cited 2022 Jun 15). https://www.phgfoundation.org/report/pathogen-genomics-into-practice.

[19] Meehan CJ, Goig GA, Kohl TA, Verboven L, Dippenaar A (2019). Whole genome sequencing of *Mycobacterium tuberculosis*: current standards and open issues. Nat Rev Microbiol.

[20] Zhou W, Wang W (2021). Fast-spreading SARS-CoV-2 variants: challenges to and new design strategies of COVID-19 vaccines. Signal Transduct Target Ther.

[21] Oude Munnink BB, Nieuwenhuijse DF, Stein M, O’Toole Á, Haverkate M (2020). Rapid SARS-CoV-2 whole-genome sequencing and analysis for informed public health decision-making in the Netherlands. Nat Med.

[22] Phan MVT, Anh PH, Cuong NV, Munnink BBO, van der Hoek L (2016). Unbiased whole-genome deep sequencing of human and porcine stool samples reveals circulation of multiple groups of rotaviruses and a putative zoonotic infection. Virus Evol.

[23] WHO (2021). SARS-CoV-2 genomic sequencing for public health goals. WHO - Interim guidance. https://www.who.int/publications-detail-redirect/WHO-2019-nCoV-genomic_sequencing-2021.1.

[24] (2022). Key actions for a united front to beat COVID-19 (Internet). https://ec.europa.eu/commission/presscorner/detail/en/ip_21_143.

[25] ECDC Expert opinion on whole genome sequencing for public health surveillance Strategy to harness whole genome sequencing to strengthen EU outbreak investigations and public health surveillance. (cited 2022 Jun 15). https://www.ecdc.europa.eu/en/publications-data/expert-opinion-whole-genome-sequencing-public-health-surveillance.

[26] Jajou R, de Neeling A, van Hunen R, de Vries G, Schimmel H (2018). Epidemiological links between tuberculosis cases identified twice as efficiently by whole genome sequencing than conventional molecular typing: a population-based study. PLoS One.

[27] Blackstone J, Stirrup O, Mapp F, Panca M, Copas A (2022). Protocol for the COG-UK hospital-onset COVID-19 infection (HOCI) multicentre interventional clinical study: evaluating the efficacy of rapid genome sequencing of SARS-CoV-2 in limiting the spread of COVID-19 in UK NHS hospitals. BMJ Open.

[28] Minh BQ, Schmidt HA, Chernomor O, Schrempf D, Woodhams MD (2020). IQ-TREE 2: new models and efficient methods for phylogenetic inference in the genomic era. Mol Biol Evol.

[29] Bouckaert R, Vaughan TG, Barido-Sottani J, Duchêne S, Fourment M (2019). BEAST 2.5: an advanced software platform for Bayesian evolutionary analysis. PLoS Comput Biol.

[30] Francis RV, Billam H, Clarke M, Yates C, Tsoleridis T (2022). The impact of real-time whole-genome sequencing in controlling healthcare-associated SARS-CoV-2 outbreaks. J Infect Dis.

[31] Brown CM, Vostok J, Johnson H, Burns M, Gharpure R (2021). Outbreak of SARS-CoV-2 infections, including COVID-19 vaccine breakthrough infections, associated with large public gatherings - Barnstable County, Massachusetts, july 2021. MMWR Morb Mortal Wkly Rep.

[32] Dorp C van, Goldberg EE, Hengartner N, Ke R, Romero-Severson EO (2021). Estimating the strength of selection for new SARS-CoV-2 variants. Nat Commun.

[33] Wolter N, Jassat W, Walaza S, Welch R, Moultrie H (2022). Early assessment of the clinical severity of the SARS-CoV-2 omicron variant in South Africa: a data linkage study. Lancet.

[34] Raman R, Patel KJ, Ranjan K (2021). COVID-19: unmasking emerging SARS-CoV-2 variants, vaccines and therapeutic strategies. Biomolecules.

[35] Weisblum Y, Schmidt F, Zhang F, DaSilva J, Poston D (2020). Escape from neutralizing antibodies by SARS-CoV-2 spike protein variants. Elife.

[36] Adapting COVID-19 vaccines to SARS-CoV-2 variants: guidance for vaccine manufacturers | European Medicines Agency (Internet). (cited 2022 May 12). https://www.ema.europa.eu/en/news/adapting-covid-19-vaccines-sars-cov-2-variants-guidance-vaccine-manufacturers.

[37] Chen J, Wang R, Gilby NB, Wei GW (2022). Omicron variant (B.1.1.529): infectivity, vaccine breakthrough, and antibody resistance. J Chem Inf Model.

[38] Corti D, Purcell LA, Snell G, Veesler D (2021). Tackling COVID-19 with neutralizing monoclonal antibodies. Cell.

[39] de Araujo CM, Guariza-Filho O, Gonçalves FM, Basso IB, Schroder AGD (2022). Front lines of the COVID-19 pandemic: what is the effectiveness of using personal protective equipment in health service environments?-a systematic review. Int Arch Occup Environ Health.

[40] Ingram C, Downey V, Roe M, Chen Y, Archibald M (2021). COVID-19 prevention and control measures in workplace settings: a rapid review and meta-analysis. Int J Environ Res Public Health.

[41] Desai AN, Aronoff DM (2020). Masks and Coronavirus Disease 2019 (COVID-19). JAMA.

[42] Ainsworth B, Miller S, Denison-Day J, Stuart B, Groot J (2021). Infection control behavior at home during the COVID-19 pandemic: observational study of a web-based behavioral intervention (Germ Defence). J Med Internet Res.

[43] Harvey WT, Carabelli AM, Jackson B, Gupta RK, Thomson EC (2021). SARS-CoV-2 variants, spike mutations and immune escape. Nat Rev Microbiol.

[44] Triggle CR, Bansal D, Ding H, Islam MM, Farag EABA (2021). A comprehensive review of viral characteristics, transmission, pathophysiology, immune response, and management of SARS-CoV-2 and COVID-19 as a basis for controlling the pandemic. Front Immunol.

[45] Hall VJ, Foulkes S, Saei A, Andrews N, Oguti B (2021). COVID-19 vaccine coverage in health-care workers in England and effectiveness of BNT162b2 mRNA vaccine against infection (SIREN): a prospective, multicentre, cohort study. Lancet.

[46] Hall VJ, Foulkes S, Charlett A, Atti A, Monk EJM (2021). SARS-CoV-2 infection rates of antibody-positive compared with antibody-negative health-care workers in England: a large, multicentre, prospective cohort study (SIREN). Lancet.

[47] Abbas M, Robalo Nunes T, Martischang R, Zingg W, Iten A (2021). Nosocomial transmission and outbreaks of coronavirus disease 2019: the need to protect both patients and healthcare workers. Antimicrob Resist Infect Control.

[48] Locke L, Dada O, Shedd JS (2021). Aerosol transmission of infectious disease and the efficacy of Personal Protective Equipment (PPE): a systematic review. J Occup Environ Med.

[49] Hodcroft EB, Zuber M, Nadeau S, Vaughan TG, Crawford KHD (2021). Spread of a SARS-CoV-2 variant through Europe in the summer of 2020. Nature.

[50] Williams GH, Llewelyn A, Brandao R, Chowdhary K, Hardisty K-M (2021). SARS-CoV-2 testing and sequencing for international arrivals reveals significant cross border transmission of high risk variants into the United Kingdom. EClinicalMedicine.

[51] Corey L, Beyrer C, Cohen MS, Michael NL, Bedford T (2021). SARS-CoV-2 variants in patients with immunosuppression. N Engl J Med.

[52] Bager P, Wohlfahrt J, Fonager J, Rasmussen M, Albertsen M (2021). Risk of hospitalisation associated with infection with SARS-CoV-2 lineage B.1.1.7 in Denmark: an observational cohort study. Lancet Infect Dis.

[53] Goldstein E, Lipsitch M, Cevik M (2021). On the effect of age on the transmission of SARS-CoV-2 in households, schools, and the community. J Infect Dis.

[54] Harper H, Burridge A, Winfield M, Finn A, Davidson A (2021). Detecting SARS-CoV-2 variants with SNP genotyping. PLoS One.

[55] Anti-SARS-CoV-2 monoclonal antibodies | COVID-19 treatment guidelines (Internet). (cited 2022 May 12). https://www.covid19treatmentguidelines.nih.gov/therapies/anti-sars-cov-2-antibody-products/anti-sars-cov-2-monoclonal-antibodies/.

[56] O’Toole Á, Hill V, Jackson B, Dewar R, Sahadeo N Genomics-informed outbreak investigations of SARS-CoV-2 using civet. Epidemiology.

[57] Campbell F, Didelot X, Fitzjohn R, Ferguson N, Cori A (2018). outbreaker2: a modular platform for outbreak reconstruction. BMC Bioinformatics.

[58] Illingworth CJR, Hamilton WL, Jackson C, Warne B, Popay A (2022). A2B-COVID: a tool for rapidly evaluating potential SARS-CoV-2 transmission events. Mol Biol Evol.

[59] Stimson J, Gardy J, Mathema B, Crudu V, Cohen T (2019). Beyond the SNP threshold: identifying outbreak clusters using inferred transmissions. Mol Biol Evol.

