# Supplementary material for: How public health authorities can use pathogen genomics in health protection practice: a consensus-building Delphi study conducted in the United Kingdom
Source: Microb Genom. 2023 Feb 6;9(2):mgen000912. doi: 10.1099/mgen.0.000912 (PMC9997744; doi:10.1099/mgen.0.000912)
Supplement: Supplementary material 1 [file mgen-9-912-s001.pdf]

Supplementary Table 1. Conducting and REporting of DElphi Studies (CREDES) checklist.

Item	Evidence
Justification	Described in the introduction.
Planning and process.	Described in methods.
Definition of consensus.	Described in methods.
Informational input.	Information provided to the expert panel was reviewed and piloted by the study team.
Prevention of bias.	The researchers took measures to avoid influencing the experts' judgements.
Interpretation and processing of results.	Described in methods
External validation.	This manuscript was submitted to the COG-UK publication reviewers for validation.
Purpose and rationale.	Described in the introduction.
Expert panel.	Described in methods.
Description of the methods.	Described in methods.
Procedure.	Described in methods.
Definition and attainment of consensus.	Described in methods.
Results.	Reported separately in Results and Supplementary Files.
Discussion of limitations.	Limitations included in discussion
Adequacy of conclusions.	The recommendations are linked to the results in the conclusions.
Publication and dissemination.	This manuscript was submitted for publication and disseminated through COG-UK channels.

How Public Health Authorities can use Pathogen Genomics in Health Protection Practice: A Consensus-building Delphi Study conducted in the United Kingdom

Survey questions

Round 1

1. How are you currently involved in SARS-CoV-2 genomics information flows?
2. What are the goals of sequencing SARS-CoV-2?
3. How should SARS-CoV-2 genomic information be used in risk assessment and management in public health practice?
4. In the context of a known cluster or outbreak of epidemiologically linked COVID-19 cases, how should SARS-CoV-2 genomic information be used? Include what type of information (e.g., sequence, lineage) and what methods should be applied (e.g., use of specific methods, tools or techniques for understanding risk).
5. How do the lineages circulating in the population affect how genomic information should be used? (e.g., in the context of near-saturation of one lineage, or the possibility of emerging variants)
6. How does the proportion of PCR-positive samples that have been sequenced affect how genomic information should be used in this context?
7. How does the timeliness of genomic results affect how genomic information should be used in this context?
8. How does the completeness or quality of sequence coverage affect how genomic information should be used in this context?
9. How does the setting of the outbreak affect how genomic information should be used? (e.g., in a relatively closed setting such as a care home, compared to an open setting such as an entertainment event.)
10. How should statistical uncertainty about transmission be considered?
11. Unsuspected transmission events between people who have COVID-19 might be elicited from comparing genomic data from unrelated isolates. Should SARS-CoV-2 genomic information be used in this way? If so, how? Include what type of information (e.g., sequence, lineage) and what methods should be applied (e.g., use of specific methods, tools or techniques for identifying transmission events).
12. How do the lineages circulating in the population affect how genomic information should be used? (e.g., in the context of near-saturation of one lineage, or the possibility of emerging variants).
13. How does the proportion of PCR-positive samples that have been sequenced affect how genomic information should be used in this context?
14. How does the timeliness of genomic results affect how genomic information should be used in this context?
15. How does the completeness or quality of sequence coverage affect how genomic information should be used in this context?
16. In what settings, if any, should public health authorities seek to identify transmission through genomic similarity?
17. How should statistical uncertainty about transmission be considered in the use of genomic information in this context?

18. How should public health authorities decide how many SARS-CoV-2 isolates should be sequenced?
19. If sequencing of all isolates is not possible, what principles should guide prioritisation of samples for sequencing?
20. Tell us any other ways in which SARS-CoV-2 sequencing should be used for risk assessment or risk management that was not covered in your answers above.
21. In a public health department or infection prevention and control team, what knowledge and skills are required to interpret genomic information to support day-to-day COVID-19 cluster and outbreak risk assessment or management?

Round 2

1. How are you currently involved in SARS-CoV-2 genomics information flows?
2. Public health authorities should use pathogen genomic sequencing to detect the emergence of new variants.
3. Public health authorities should use pathogen genomic sequencing as a surveillance tool to monitor geographical spread of variants over time.
4. Public health authorities and healthcare providers should use pathogen genomic sequencing in outbreak investigations in health care settings to rule in or rule out transmission events.
5. Public health authorities should use pathogen genomic data to inform estimates of epidemic growth.
6. Public health authorities should use pathogen genomic data to inform estimates of clinical severity of the disease
7. Public health authorities should use pathogen genomic data to detect changes in risk of infection in specific settings e.g., schools, care-homes etc.
8. Pathogen genomic information should inform evaluation of the effect of:
    - non-pharmaceutical interventions
    - vaccines
    - pharmaceutical therapeutic treatments
9. Pathogen genomic information should be linked to contextual epidemiological information to facilitate risk assessment.
10. Public health authorities should prioritise sequencing from outbreaks where it appears there is greater than expected disease severity.
11. Public health authorities should sequence enough randomly selected samples to enable unbiased surveillance
12. Public health authorities should prioritise sequencing of samples from vaccinated people.
13. Public health authorities should prioritise sequencing of samples from people who have had multiple episodes of infection.
14. If it is not possible to sequence all samples, public health authorities should direct sequencing capacity towards vulnerable populations.

15. If it is not possible to sequence all samples, public health authorities should direct sequencing capacity towards outbreak investigation.
16. If it is not possible to sequence all samples, public health authorities should direct sequencing capacity towards populations in whom new variants might be present e.g., international travellers and immunocompromised people.
17. Public health authorities should use pathogen genomic data to de-escalate potential outbreaks that were identified through epidemiological links.
18. Timeliness of genomic sequencing results is important to allow results to be acted upon by public health authorities.
19. Clinical teams need timely access to sequence results to inform treatment and infection control decisions.
20. A minimum of ten percent of all positive covid-19 samples should be sequenced.
21. Public health authorities should use tools such as rapid genotyping for surveillance of lineages.
22. Public health authorities should analyse sequence and/or single nucleotide polymorphism data as part of investigation of outbreaks.
23. Analysts should exclude sequences below a defined sequence coverage from analysis when investigating transmission events.
24. Public health authorities should use bioinformatics tools to elicit unsuspected transmission events.
25. Public health authorities should ensure training is provided for health protection, infection control and clinical teams on the interpretation of sequencing results.

Round 3

1. How are you currently involved in SARS-CoV-2 genomics information flows?
2. If it is not possible to sequence all samples, public health authorities should direct sequencing capacity towards people who are at greater risk of adverse outcomes from infection.
3. If it is not possible to sequence all samples, public health authorities should direct sequencing capacity towards outbreak investigations:
    - In closed settings, such as care homes and hospitals.
    - In community outbreaks, such as at public events or functions.
4. Public health authorities should use pathogen genomic data to de-escalate potential outbreaks that were identified through epidemiological links:
    - In closed settings, such as care homes and hospitals.
    - In community outbreaks, such as at public events or functions.
5. Pathogen genomic information should inform evaluation of the effect of non-pharmaceutical interventions.
6. The proportion of samples sequenced should reflect the epidemiological context.

7. Public health authorities should analyse sequencing data in more detail than lineage as part of the investigation of outbreaks.
8. Analysts should only include sequences above defined sequence coverage depth when investigating transmission events. (This is an overall quality control value provided for all sequencing results).

Survey Responses

Information that reveals the region or organisation of the respondent has been removed, as have the names of individual experts mentioned by respondents. Non-response answers (e.g. “I do not know”) have been removed.

Round One

**Q2. What are the goals of sequencing SARS-CoV-2?**
Surveillance - detection of new variants and monitoring their spread; use in outbreak investigations especially in health care settings to clarify epidemiological links between cases
- Tracking and tracing the spread of new variants - Predicting the impact of mutations on the virus (in terms of infectivity, virulence, vaccine evasion etc.) - Estimating the mutation rate
point source of outbreaks new variant detection differences with other nations
To understand the transmission, evolution and biology of SARS-CoV-2 to improve the public health response to COVID-19, by preventing of SARS-CoV-2 infections and improving treatment of COVID-19 disease.
To identify variants, both the prevalence of known variants within the population and detection of novel variants
1. Public health surveillance - monitor the circulating variant / strain of virus 2. Outbreak analysis 3. Support & monitoring of direct patient care - i.e. during therapeutic treatment
To be aware of which variants are circulating, their prevalence and likely transmission routes. This information is required to inform public health action (lockdowns/testing policies/vaccination priorities/school closures, etc). Sequencing provides contextual information to cluster and outbreaks to aid guidance. Sequencing can be used alongside information about disease severity and vaccination status to learn about the profiles of each variant.
Identify variants of concern Elucidate patterns of transmission (although this has been challenging in our study because of poor sequencing coverage)
To identify, risk assess and track the spread of variant strains. To identify routes of transmission in the context of outbreaks. To gain a better understanding of the virus.
Understanding transmission dynamics for example in an outbreak. SIngle or multiple seeding. Development of variants and there changing characteristics. Severity, transmissability and immune escape.
To inform how transmission occurs To inform policy about public health interventions To help understand local outbreaks and provide subsequent interventions
Cluster outbreak identification at local and national levels, surveillance for novel and of identified variants of concern, tracking geographical migration (including introduction) of SARS-CoV-2 into a country, assessment of vaccine efficacy, assist development of diagnostics and therapeutics, identify cases of reinfection, identify zoonotic transmission.
To look at: the emergence of new variants; the importation of new variants; the spread of variants across [area name]; the spread of variants within specific locations e.g. care homes.
Outbreak investigation Understanding and identifying new variants Developing WGS as a service for other pathogens

**Q3. How should SARS-CoV-2 genomic information be used in risk assessment and management in public health practice?**
Identification of new variants linked to international travel, assessing transmission of new variants, assessing epidemiological links between cases to investigate outbreaks
- For monitoring of appearance and spread of new variants - For prediction of the potential effect of mutations on the efficiency of vaccines, antivirals, and on biological characteristics of the virus
WGS important in early response to new variants. There can be a delay in the development of genotype assays for new variants eg recent experience with omicron where assay in pillar 2 was not available for c 10 days and WGS was crucial. In outbreak investigation; sublineages. For HCAIs this is important to check if cases are linked. For families it helps them understand what has happened. Alpha wave HCAIs had a high mortality compared to subsequent waves.
It should be used day-to-day to understand the population of SARS-CoV-2 to enable: (1) monitoring the emergence of variants of concern, (2) identify mutations / variants with altered biological properties, (3) to better understand the importation and transmission of SARS-CoV-2 to improve infection control.
Monitoring for previously defined 'variants of concern' , detection of which can be used to manage specific outbreaks. Analysis of individual mutations to support identification of transmission chains. Knowledge of these routes can be used to inform practice. Early detection of novel variants with either predicted characteristics of concern (mutations) or displaying high transmission rates or more severe disease.
Surveillance of circulating variants informs analysis on: - reproduction number - clinical severity - mechanism of transmission & risk e.g. schools - impact of Non Pharmaceutical Interventions - vaccine effectiveness - therapeutic effectiveness
Genomic information should be used alongside available information regarding disease severity and immune escape to advise on target groups for vaccination. This information should also be used to advise on outbreak behaviour (self isolation/visitors allowed, etc) based on the determined risk to specific groups (elderly/infants/immune compromised, etc)
Monitor variants to inform decisions about the need for re-vaccination / likely protective effect of current vaccines Potential use to investigate outbreaks and clusters of infection beyond the pandemic in closed settings e.g. care homes, hospitals
I feel this information is most useful in conjunction with other information. Eg: it can be used to compare emerging virus strains with other circulating strains in terms of transmissibility and severity in order to identify VOCs and VUIs. It can be used with epi information during outbreaks to identify sources of the outbreak and transmission chains.
Identifying and confiring transmission pathways
Allows understanding of where transmission is occurring in order to inform 1. policy 2. localised management of specific outbreaks
SARS-CoV-2 genomic information should be critical to risk assessment and management in public health practice. In combination with detailed epidemiological information, sequencing can improve granularity of information significantly. It is vital to understanding growth rates of VOCs, in understanding transmission dynamics in a given geographical location, and highlighting possible scenarios where vaccine efficacy is compromised.
To enable the points in Qu2
Due to time lag - no current utility in outbreak control measures. Should be used to inform PHresponse to new variants, by targeted sequencing of samples of interest. PH resopnse would be regionally implemented control measures

**Q4. In the context of a known cluster or outbreak of epidemiologically linked COVID-19 cases, how should SARS-CoV-2 genomic information be used? Include what type of information (e.g. sequence, lineage) and what methods should be applied (e.g. use of specific methods, tools or techniques for understanding risk).**
It depends on the context. If the outbreak is in a high risk setting such as a hospital lineage as well as sequence is important to differentiate multiple sources and exposures. [region-specific sentence censored]. In other less high risk settings it is sometimes useful to see if a large outbreak in a workplace or entertainment venue is associated with a single source or multiple seeding. Sequence can be useful at the level of household clusters for example when tracking the introduction of new VOCs/VUIs in the context of international travel.
To date I have used for lineages and outbreak investigations. The info is available [region-specific information censored]. Limited experience in Trusts of use of WGS in outbreak investigation.
Genomics should be used to supplement good quality epidemiological data. A combination of phylogenetic analysis paired with epidemiological data should be used as the gold standard.
Viral sequences could be used to contribute to definition of transmission chains
[names censored] should comment. Analysis requires genomic data LINKED to information on (i) location (ii) sampling date (iii) vaccination status (iv) contact tracing info inc known exposures (v) travel information (vi) clinical severity / hospital admissions
In a known cluster or outbreak, a minimum of lineage information should be used to determine linked cases. A lineage is defined as any sequence containing an allowed set of mutations, therefore is heterogeneous by its very nature. It would be valuable to use analysis tools that could identify common patterns in sequences across cases in a cluster. This could be used to identify clusters within a cluster to help determine the index case(s) and possible mode of transmission.
estimate the likelihood and direction of transmission between individuals (sequence) Usage of tools to infer probability of transmission such as those developed in the HOCI study This requires integration of sequencing and epidemiological data
I feel this info is very useful in the context of clusters/outbreaks. Very useful is the SNP address for identification of closely related viral strains, giving information about transmission.
Two levels. Wider genotype at population level. More detailed lineage when investigating specific transmission in time place and person (eg [specific outbreak detail censored])
They should answer the question "How likely is it that these 2 individuals acquired COVID from one another"? I dont have an understanding of each of the methodologies to answer the second part.
Lineage and sequence information are both useful in understanding a known cluster or outbreak of COVID-19 cases. Lineage information may crudely provide certainty to the idea linked individuals are within a transmission cluster - though this information has become less useful once certain lineages (alpha, delta, omicron) have gained a transmission advantage. Various other methods of providing certainty to whether a cluster of cases are indeed linked including civet (focus on phylogenetic linkage within a defined number of nodes) and A2B-COVID (providing context with timing, sequence, and possibly location information). Examination on a maximum likelihood phylogenetic tree, at least a local level, and examined in a tool like microreact can provide an interact assessment of the outbreak. Further detail can be provide with a time-scaled tree (built in IQTREE-2 or BEAST), or by examination of sequences within a very limited SNP threshold, such as 'zero-SNPS' to examine relationships between categories of individuals (such as between university households, courses, or healthcare workers and patients).
To confirm the epidemiological links. WGS will give the information required to confirm [or not] that patient isolates are the same.

**Q5. How do the lineages circulating in the population affect how genomic information should be used? (e.g. in the context of near-saturation of one lineage, or the possibility of emerging variants)**
At an early stage of introduction we are keen to know the WGS for a high proportion of cases. There is a tipping point where genomic information becomes less critical - certainly above 50%. Detection of new variants/mutations remains an important surveillance function
In the context of near-saturation of one lineage genomic sequencing should be maintained at a level that would allow to detect early appearance of new variants
The emergence of a clear dominate lineage will limit the use of lineages, etc for epidemiological purposes. But the use of phylogenetics paired with epidemiological data will still be of merit.
If there is near saturation with one lineage genomic information provides the only opportunity to track transmission through individual mutations. Sequencing is perhaps most valuable when a new variant is emerging and strain-specific RT-PCR assays are not available. Rapid turnaround sequencing from individuals of interest is then valuable.
In the case of a dominant lineage, genomic information should be used to analyse characteristics of the lineage, for example speed of transmission and vaccine evasion. These findings should be compared to previous dominant lineages, along with other countries' findings to improve understanding of how specific mutations may relate to specific characteristics of the virus. Genomic surveillance should be used to identify new variants and used alongside information about travel to determine the source. In an ideal world, screening in this way could be used to prevent introduction of new variants, but more likely it will allow public health measures to slow the spread while more information on variant risk is gathered. Genomic information on variants should be used to determine the ability of new variants to out-compete existing variants. This will allow mutation profiles with associated behaviours to be identified, which can be used for future screening, i.e. high transmission plus high disease severity plus high vaccine evasion. If key genomic profiles could be used as accurate predictors of virus behaviour, this would be a very important public health tool to enable appropriate response measures.
If there is a simple marker that correlates with a variant e.g. Omicron and SGTF there is an argument that less sequencing might be required, but there is the persistent risk that new variants will emerge that can only be identified by sequencing.
I think this context is always important to have in mind when interpreting SARS-CoV-2 genomics info. I still think there is value in the this info even in the saturation scenario. It may be helpful to see if a tipping point occurs and as mentioned useful to identify emerging strains.
At populationlevel mapping temporospatial distribution over parts o fthe region
Lineage can only provide the answer "these 2 individuals were unlikel to aqcuired COVID from one another". This may not therefore be very helpful.
A diverse group of circulating lineages was easier to work with. Though I have not formally examined this myself, I imagine a faster growth rate of lineages makes detection of epidemiologically linked cases more difficult (with fewer opportunities for SNP-differences to build between cases). That said, using a phylogenetic approach (such as that provided by civet) can still prove helpful in demonstrating when cases are not linked. At a wider level, the emergence of a lineage (and possible VOC) may impact targeted surveillance and direct utilisation of data in a different way (assessment of importations, vaccine efficacy, development of therapeutics, etc).
It is important to know the lineages circulating in the community. Once the threshold [90%] is reached then the numbers submitted for WGS can be reduced. However, it is still important to keep monitoring community isolates for the emergence of any new lineages or variants.

**Q6. How does the proportion of PCR-positive samples that have been sequenced affect how genomic information should be used in this context?**
The proportion of positive samples that are sequenced and the sampling strategy will affect the confidence which can be placed on the results.
We need WGS for surveillance where a sample of positives in required and outbreak investigation where all should be tested if technically feasible.
The proportion of cases tells you how likely (in simple terms) that your sequencing data is representative of the viral population. If you are sequencing 10% of cases at random and getting all the severe cases you can have a certain degree of confidence that you are seeing capturing a reflective snapshot . But of course you aren't seeing what is going on in 90% of the community samples - which means it will probably take longer for you to see the emergence of a novel lineage.
If the proportion of samples sequenced falls too low then this is no longer an effective approach to detect variants of concern. In terms of modelling the population dynamics of variants, the lower the proportion the samples sequenced the more uncertainty there will be - the appropriate cut-off being a question for statisticians!
A higher proportion of PCR positive sequencing provides a larger and more complete dataset to analyse patterns. This allows creation of more accurate models of, for example, mutation profiles versus disease severity. A smaller proportion of PCR positive sample sequencing reduces the statistical power of any analysis. Sampling in this way lends itself better to surveillance; to get a picture of circulating variants and their relative proportions. However, smaller proportion sequencing makes it more likely that emerging variants will not be detected until they have already entered the community transmission stage.
It depends on which populations are prioritised for sequencing. It may be important to sequence a higher proportion of samples from high-risk populations if variants are more likely to emerge in these groups e.g. immunocompromised patients
Again I think this context is very important and should be taken into account when interpreting genomics results. In a period of low proportions sequenced caution is necessary but the information is always useful.
Specific situations 100% if possible but surveillance a significant sample
This may allow policy to be determined ie transmissions are occurring in schools vs in restaurants. It probably isnt useful in ward level questions
Regardless of circulating lineages, fewer sequences will increase the chance of missed transmission chains and therefore potentially skew findings of an outbreak investigation (e.g. demonstrate a lack of transmission, when there was). Further, in the context of a highly transmissible lineage, SNP differences between cases ascertained through increased sequencing may be of greater use to identify transmission dynamics. Having a consistently high proportion of sequenced genomes also allows us to accurately understand how the variants are emerging.
Not sure about this. Is this about the prioritisation of samples? The greater the number of samples sequenced in an outbreak or cluster, the more reliable the epidemiology.

**Q7. How does the timeliness of genomic results affect how genomic information should be used in this context?**
For acute health protection response timeliness is very important and in this context the use of reflex assays has been extremely important so that the information can inform risk assessment and decision making. For surveillance and descriptive epidemiological purposes timeliness is less critical.
Depends on new variants and trajectory of cases associated with new variants. Ideally for outbreaks a result in 72 hours could be helpful. Surveillance results can take longer.
Timelines is critical to provide an up to date snap shot of the viral population. The longer the time delay the less current the picture of the leading edge of the pandemic. This can mean that the data is well out of date and so can't be acted upon in the same way. (e.g. the ship has sailed)

Rapid turnaround most important when detecting/confirming new variants of concern as they appear
[[names censored] should comment] but sequence data has a lag of 5-10 days which represents a significant delay when dealing with a rapidly expanding epidemic wave
Slower genomic sequencing is less useful for public health action; this would be more useful for retrospective surveillance. Faster sequencing allows for rapid identification of new variants, and therefore responses (e.g. school closure) are more likely to have an effect on the spread of the variant.
Timeliness has a major impact on how the data can be used. If the goal is to inform outbreak investigations then the data need to be available in near real-time. Monitoring variants also requires data to be available on short time scales. Timeliness is less relevant for research studies that use sequencing to gain insight into patterns of transmission
I think this is very important in an outbreak scenario in terms of timely public health action. The closer to real-time is obviously better. I think a greater delay can be tolerated for surveillance purposes but in the pandemic scenario I think this delay has more consequences than during an endemic situation. The more timely the better.
Timeliness where there is an expected decision point in public health implementation. Manage the future and then understand the past.
In hospitals it would be helpful retrospectively to see where transmissions are occurring ie this occured in the ED vs the ward vs the community
Timeliness of genomic information is critical. Identifying the introduction and dispersal of a lineage in a hospital for example can provide vital information to the necessary IPC measures. At a national level, this information can be important in understanding the importations of a lineage. As the pandemic progresses, the goal of understanding the introduction and spread of a lineage is likely to remain important; more sinister variants of concern could emerge and having effective mitigation strategies is important - timing of genomic information is critical to this. Timing of information is also critical to assessing growth rates of a lineage.
Important particularly in the early phases of a cluster or when considering the treatment of individual patients. TATs could be influenced by the clinical or epidemiological need.

**Q8. How does the completeness or quality of sequence coverage affect how genomic information should be used in this context?**
The completeness or quality of coverage of positive samples will affect the confidence which can be placed on the results and therefore the usefulness of the information for risk assessment and decision-making.
Poor quality sequencing data should not be used as it can not be relied upon. It certainly can't be used for phylogenetic analysis which should be used as the gold standard for molecular epidemiology. Poor coverage data might be used (if it must) to infer a lineage - though there are risks with this.
Current QC seems appropriate for confident lineage calling. Useful information could potentially be extracted from lower quality samples in rare specific cases of particular interest
Quality & coverage are important - particularly in specific regions of the genome which are evolving rapidly or are selected through vaccine / therapeutic interventions.
Higher quality sequence coverage improves the power of any analysis, making any associations to viral characteristics more robust. This makes it easier to have clear policies in place for public health action, which are proportionate to the risk of the variant. With lower quality sequence coverage, the risk of a new variant will be less well understood so sweeping measures may need to be provided while intelligence is gathered.
If a substantial proportion of samples are not sequenced because of high Ct values this may impact on the ability to reconstruct transmission chains. In the context of an outbreak, if samples are only sequenced for a small proportion of exposed cases this may bias conclusions that are drawn about transmission.

I think the higher quality the sequences the better but some information is better than none at all.
Technical issue and look to virologist for advice in interpretation
Probably doesnt matter if not very complete
Variable sequence coverage makes understanding transmission very difficult at a national and local scale. For the purpose of vaccine efficacy, diagnostic and therapeutic development, coverage does not impact greatly - the lineages will eventually be identified if concerning. For outbreak investigation, or for comparing populations, or transmission between populations, conclusions are highly limited by poorly complete or variable sequence coverage.
Reflex or assays such as SGTF can be used when the prevalence of an isolate is high e.g. omicron otherwise the complete sequence is important e.g. in spotting a new variant.

**Q9. How does the setting of the outbreak affect how genomic information should be used? (e.g. in a relatively closed setting such as a care home, compared to an open setting such as an entertainment event.)**
f the outbreak is in a high risk setting such as a care home lineage as well as sequence is important to differentiate multiple sources and exposures. For example at the present time with high community prevalence many of the clusters in care homes are associated with staff cases related to transmission outside the care home. In other less high risk settings it is sometimes useful to see if a large outbreak in a workplace or entertainment venue is associated with a single source or multiple seeding. For most outbreaks these settings we would not look for WGS on each possible case.
Depends on associated mortality and vulnerability of exposed population. Alpha HCAIs had a c30% mortality, Delta HCAIs had 20% mortality. Omicron looks like having a lower mortality rate but early days.
The quality of the epidemiological data is really the key issue. In a care home this might be good as good records will exist for who met who, which locations they went, etc. In a large venue the best that will likely be possible is they attended an event or sat in a different area. Investigations of both can be improved by genomics data - but the granularity of the answers will differ.
Sequence level analysis beyond lineage likely to be required in a closed setting
In open settings, genomic information can be used as a screening tool to identify the sources of different mutation profiles and to get a view of the potential methods of transmission. In closed settings, genomic information should be more complete (i.e. whole home sequencing) and could be used to identify the source of the outbreak. Genomic information could also be used to determine appropriate measures, for example an outbreak of the dominant lineage may not warrant additional measures, whereas outbreak in a closed setting of a high risk variant would require stricter restrictions, enhanced cleaning etc.
It depends on the public health actions that will be taken on the basis of the sequencing data. It is likely to be more important to prevent outbreaks in vulnerable populations such as care homes. The risk/benefits of contact tracing after a large event attended by the (mainly healthy) general public may be limited if severe outcomes following infection are rare.
I think the information is more useful in an open setting when there is likely to be more heterogeneity of SARS-CoV-2 strains and potentially multiple sources of transmission.
A well defined population is easier to study than a popuation linked to an event or venue
More helpful where the epidemiological data would support some of the cases likely epidemiologically to have acquired it elsewhere ie CARE home more likey to be an outbreak in that geography whereas cinema more likely aqcuired elsewhere
The setting of an outbreak is important. Not only is the closed or open nature important, but also the population of the setting. We know once a lineage is introduced into a care home, subsequent cases in such a setting are often the same lineage. Therefore, further genomic information may not be useful, with a baseline surveillance sufficient. In a hospital however, we see a far more open setting

with introductions possible from the community at a more regular interval and spread to wards from outpatient clinics, staff, between wards, etc. Here, a more consistent and higher coverage of sequencing may be of use to identify problematic areas and implement IPC measures earlier. Social settings are less well understood but detailed contact tracing, above genomic information appears more useful. Social facilities are possible sources of dispersal of SARS-CoV-2, drivers for onward transmission (rather than persistent transmission, such as in a care home). Here, contact tracing is essential for linking genomically linked community or other disparate cases (such as across a university) to a social venue. This can then allow for targeted IPC measures for the social venue (ventilation, reinforcing mask wearing rules, hand hygiene, etc). Conversely, genomic information is very useful in all settings when contact tracing falsely links transmission chains i.e. possibility of transmission is refuted by phylogenetic variation - other than in the context of a study, timely understanding of this is important to help de-escalate an outbreak or understand the context of an outbreak in a useful way.
More important where vulnerable people are involved e.g. in a care home or in a crucial factory or service supplier situation e.g. bus, lorry or train drivers

**Q10. How should statistical uncertainty about transmission be considered?**
Not sure, depends on stage of the pandemic wave
All molecular epidemiology is only as good as the data collected. Even with clear cut genomics data (e.g. person's A strain was ancestral to person B's strain) - caution should always be excised. Determination of transmission pathways is in essence a search for the most parsimonious explanation - so alternatives always exist but we plum for the simplest explanation.
Models should not try to fit the data too closely, to allow for this uncertainty.
Ideally this should be quantified and reported in a way that is accessible to people who have limited understanding of sequencing data. It may be useful to agree thresholds so that events can be classified as transmission likely or unlikely
Level of certainty for PH action may be less than proving something in the past for acceptance as knowledge for future situations
This is problematic but the most reliable way (and practical way) is for a concerted effort to combine genomic and epidemiological information. This requires joined up working of individuals across departments in public health agencies. Refuting transmission through genomic information by demonstrating phylogenetic variation is least problematic. Demonstrating a cluster is truly linked requires additional information such as context of the common exposure (transient visits to a supermarket may appear linked but probably aren't), community context (there may be a expansion of a unique lineage through epidemiological interactions), timing of infection (is this a chain of common exposures which are within a time-frame of plausible incubation periods, serial intervals, etc).
Important in deciding whether the event is 'real' or not.

**Q11. Unsuspected transmission events between people who have COVID-19 might be elicited from comparing genomic data from unrelated isolates. Should SARS-CoV-2 genomic information be used in this way? If so, how? Include what type of information (e.g. sequence, lineage) and what methods should be applied (e.g. use of specific methods, tools or techniques for identifying transmission events).**
At the current high incidence resources for follow up and investigation of this sort are stretched. This could be used in high risk settings such as health care.
Unsuspected transmission events should have WGS where impact is high eg death or hospitalisation. I cannot comment on the method

This is worthwhile - but will only be possible with good quality epidemiological (e.g. contact tracing) data. Approaches that take into consideration phylogeny will be essential along with the known epidemiological data. But sampling coverage and upsampled individuals must always be kept in mind (e.g. there might be important links / details missing).
Yes, because this could provide insights into previously unrecognised patterns of spread
depends on - level of sequence coverage in population - level of testing in outbreak setting - genetic diversity / evolution rate of current variant
When looking at transmission events, sequencing information should be used alongside available contextual information (shared events, job site, etc). There should always be a degree of error associated with any transmission event, particularly given the genetic variability of the virus. Commonalities across sequences should be used to derive a probability for each transmission event. Genomic information should only be used to provide additional insight into transmission events, and should not be used as the ground truth.
I think this can be justified in health or social care settings because it is important to understand how infection control practices are failing. In the community, my view is that this can only be justified if infection is strongly associated with severe outcomes, and contact tracing is necessary to protect the individuals concerned.
I think this information is useful and important and can reveal unexpected insights. I think the SNP addresses can be used to identify clusters at a regional as well as local level which may prompt further investigation.
Yes, when rates in the community are lower than current and targeted intervention is possible
Genomic information is useful at shining at light on possible transmission events. If conducted for public health purposes or under an ethically approved study, this seems like a reasonable endeavour. Without the use of epidemiological information (either through prospective contact tracing or targeted evaluation of linked clusters), the genomic information is of little use in this context. Cluster detection at this level is best through evaluation of phylogenetic (maximum likelihood tree or a tool such as civet), or use of short SNP distances (zero) determined through cluster picker or snp-dist, or with genomic information combined with timing data (A2B COVID or transcluster). Lineage information is not sufficient for this purpose.
Yes as it will enable a targeting of epidemiological efforts. Don't know enough about the type of information or the methods to comment but know people who do!

**Q12. How do the lineages circulating in the population affect how genomic information should be used? (e.g. in the context of near-saturation of one lineage, or the possibility of emerging variants).**
SUrveillance Outbreak control and surveillance Analysing and understanding the different experience (cases, hospitalisations and deaths) of the pandemic between regions and nations. Was this because the disease was milder, did we lock down early enough in the first wave, lower vaccination levels, access to new therapies, IPC procedures including appropriate use of PPE.
Multiple divergent lineages will likely make the task easier (as seen in wave 1) (e.g. you can use lineage), while homogenous since lineages will require detailed analysis using the full length of the genome. But In all cases - phylogenetic analysis is critical to get the most out of genomic data.
When looking at dominant lineages, it will be necessary to use more granular information (sequences rather than lineages) to identify patterns and determine transmission events. However, if a variant is dominant it is likely there is more genetic variance across the country, which may mean that the statistical model will need to be more forgiving of noise in the data. When looking at emerging variants, it is more likely that lineage (rather than full sequence) will be sufficient to predict transmission events.
If a lineage becomes dominant it may make sense to focus sequencing on high risk population e.g. travellers, immunocompromised to monitor for the emergence of variants?

Again this context is important but the information can still be useful in terms of observing changes in the circulation of lineages and identifying the emergence of new ones.
If one lineage predominates it will be the odd ones out to watch (VUI and VOC). Monitor risky settings like haematology oncology and HIV populations
I dont think lineages are helpful here particularly when intervention is limited
Is this about thresholds? See answer to previous question. There must be a capacity to recognise the emergence of variants even with near saturation of one lineage.

**Q13. How does the proportion of PCR-positive samples that have been sequenced affect how genomic information should be used in this context?**
statistician may be able to answer this
In short, if coverage is low then you always have a higher risk you missed something at low frequency or you missed an important sample(s) that would explain transmission.
The proportion of PCR positive samples affects the probability of any transmission event determined by the statistical model. Lower proportion of sampling may mean than transmission events are missed, and therefore inference is required, which introduces error. Higher proportion of sampling allows for higher statistical certainty of transmission events.
Depends on the target population and the public health question that is being posed.
Again, when the % of samples sequenced is low caution any information derived in this context needs to be taken with a pinch of salt and interpreted with caution.
Random samples and purposive samples needed

**Q14. How does the timeliness of genomic results affect how genomic information should be used in this context?**
Again - the closer to the leading edge of transmission the more actionable that data will be. For example if you flag a night club as being hot spot for transmission on a Thursday after a cluster of cases from the previous weekend - you would have chance to intervene. If turn around was slow it might take another week - allowing transmission the following weekend.
Rapid genomic results allows for action to be taken to prevent further transmission events. In this way, rapid genomic results enables acute response. Slower results cannot be used in this way; they can instead be used to retrospectively analyse transmission events to predict transmission behaviour at future events. In the absence of acute response action, retrospective analysis allows for more thorough modelling of patterns, which may enable faster acute response for future events.
Public health action requires timely information whereas research studies of transmission are less dependent on timely data
As before I think timeliness is always important but perhaps less so in this situation in comparison with an acute outbreak situation.
Tmeliness for managing the future PH actions.
Timliness useful if targeted intervention is possible
Again important dependent on the context.

**Q15. How does the completeness or quality of sequence coverage affect how genomic information should be used in this context?**
For fine scale phylogenetics - you need high quality genomes.
Incomplete sequences may need to be removed from analysis as they could lead to incorrect transmission events. Higher quality sequencing allows for higher statistical power when determining transmission events.

This may impact on the ability to investigate transmission events
Completeness of the sequence is also important but again perhaps less so in this scenario compared with the acute outbreak situation.
Horses for courses. Only use high quality infrmation when it is really needed. Accept trades off.
When low levels completeness is more important

**Q16. In what settings, if any, should public health authorities seek to identify transmission through genomic similarity?**
As in previous answers high risk settings principally health and social care settings.
HCAIs Outbreaks with High Attack Rates Outbreaks associated with high morbidity (hospitalisations) and deaths Outbreaks in vulnerable group
The potential has been clearly demonstrated in care homes, hospitals, planes, universities and various other settings. In short anywhere you want to learn about transmission pathways.
All settings
Identification of transmission through genomic similarity should be used with caution, and ideally with additional contextual information. It would be most useful in the case of new variants that are not known to be in community transmission.
Health and social care outbreaks to identify failure of infection control practice
I think it could be best employed in settings such as workplace and school settings where lessons can be learned for the future to inform prevention measures and to assess the effectiveness of measures already in place.
Much the same as we use phage typing and WGS in bacterial outbreaks. Was it a superspreader event?
Places where mortality is high eg hospitals and intervention (ie shutting) is possible.
In my opinion, as we still have a lot to understand about the transmission dynamics of SARS-CoV-2 at a local level, we should seek to identify transmission in many settings.   Firstly, to protect vulnerable populations (priority), transmission should be identified in care homes and hospitals settings (patient movements or staff, for example, may link otherwise unlinked wards - this can be confirmed with epidemiological information).   Though more difficult, strong associations of clusters, or growth of clusters, linked to community social venues can highlight sources of ongoing/high-risk transmission settings, such as nightclubs and schools.
The investigations of clusters or outbreaks. When linked to shoe leather epidemiology, it is extremely important.

**Q17. How should statistical uncertainty about transmission be considered in the use of genomic information in this context?**
The same as before. It is always about building the most possible situation that matches the genomics and epidemiology.
To inform overall confidence in the model of the outbreak
Transmission events should always be displayed as a probability, to highlight the uncertainty. Genomic information should be used as supporting evidence in this context, rather than the absolute truth.
This needs to be communicated to front-line practitioners in a way that is accessible
Number of snips of difference that suggest evolution rather than a second transmission route
Don't know but suspect it is most important.

Q18. How should public health authorities decide how many SARS-CoV-2 isolates should be sequenced?
PHAs need to balance lab capacity and capability, cost and opportunity cost as well as the usefulness of the information and critically whether it will be used to develop policy or make public health risk assessments.
Different for surveillance and outbreak investigation Outbreaks with significant morbidity WGS all positives Pre agree with statistician the proportion of positives that should be subject to surveillance. However each wave of pandemic was different and the severity of disease was not always ascertained for some time.
The WHO / ECDC have clear guidelines. But in short organisations should be aiming for a minimum of 10%. Mathematical modelling can be used to estimate the risks of various coverage levels.
Some sequencing capability essential. At least minimal percentage to model of lineage frequencies in the population. Exact number determined by funding available. Also consider flexibility in commissioning of sequencing to enable most appropriate service for current conditions. Different platforms are available to provide small number rapid sequences OR high throughput surveillance OR extremely high throughput for very large numbers.
depends on capacity and funding modelling can be performed to predict the sensitivity of surveillance systems under different testing thresholds
Risk of potential new variants should be considered alongside lab capacity and likelihood of change in public health response to the sequencing information.
Depends on the severity of infection and how it is impacting on society. This should consider the needs of vulnerable populations (and the risk of outbreaks in these settings) and the need to track VOCs
Ideally all samples should be sequenced if possible but this is understandably not feasible. For surveillance purposes I think if possible a representative sample of the [area name] population should be selected to ensure any info taken from the results are as generalizable as possible. That aside I think samples or work needed to inform outbreak control and/or policy decisions should take priority.
1.Surveillance and tie into UK and ROI/ECDC systems. 2.Outbreak investigation following best practice in epidemiology
The lower the community rates the more percentage.   While rationing needed, places where importance of acquiring disease or volume of spread is high
Higher case ascertainment and sequencing inevitably results in more granular detail. Transmission chains in hospital wards and care home still need to be interrupted - prioritising based on a population basis may be of use for genomic epidemiological/IPC purposes.   Identification and sequencing of reinfections should be prioritised to inform our ongoing understanding of vaccine efficacy (especially with variation across VOCs).   If the aim is to interrupt community transmission, ongoing contact tracing with sequencing of linked clusters may be a method to prioritise community isolates. If this is not feasible, background surveillance for emerging VOCs and subsequent targeted sequencing of areas of concern may be a better solution (though this would inevitably skew nationwide analyses of transmission dynamics).
This is a statistical issue and depends on the incidence in the community, locality etc.

Q19. If sequencing of all isolates is not possible, what principles should guide prioritisation of samples for sequencing?
Priority should be given to specimens from high risk settings such as health care or cases with identified risk factors - travel history, or clinical features such as immunosuppression.
Vulnerable groups: Care homes Pregnancy Immunosuppressed Children and adults with special needs People on disease modifying drugs
1. Random sampling of the population - sequenced as fast as possible this should always be the priority, 2. Of equal priority - Severe cases (ICU, hospitalised). These two should always get the

greatest priority. I would recommend then having flexible capacity to for other research/clinical priorities - e.g. vaccine failures, clinical trials, treatment failures (antivirals, etc), other research, imports.
In early stages of spread of new variant individuals at risk (eg travel-related) should be prioritised. Sufficient random samples should be selected to enable unbiassed surveillance.
Protection of key risk areas/groups. For example, if a new variant is introduced, sequencing of cases associated with high risk travel should be prioritised to reduce the chance of community transmission. Once community spread has occurred, prioritisation of cases in hospitals and carehomes should take priority since these are likely to be the patients with highest risk to health from increased disease severity/immune escape.
It should be guided by priority public health questions that the sequencing data will be used to address. For example if monitoring VOCs is the priority, then sampling should focus on the population subgroups that are most likely to acquire VOCs e.g travellers, immunocompromised. If the goal is to prevent outbreaks in high-risk settings, then the priority is good sequencing coverage in these locations.
As above I think outbreak control and/or information needed for policy decisions should be prioritised.
As above. Surveillance with comparable systems with random samplng. Understanding transmission in outbreaks in closed an other settings
1. Death rate high in cases 2. Volume of transmitted cases is high
Priority populations (hospitals, schools), community surveillance for exceedance detection (possibly epidemiological or VOC linked which can then be investigated for cause), importations for VOC surveillance, reinfections.
Statistics again. The clinical or circumstantial importance as previously.

**Q20. Tell us any other ways in which SARS-CoV-2 sequencing should be used for risk assessment or risk management that was not covered in your answers above.**
Imports, severity of disease.
In the future, sequencing information could be used to plan for vaccine manufacturing/vaccine programmes - for example, if key mutation patterns or key age group transmission patterns are established.
Risk of re-infection and duration of viral shedding with different variants
Chance findings of interest warranting epidemiologicla investigation
In an ideal world with adequate screening and sequencing - transmission chains would be more easily interrupted not only through self-reported symptomatic cases but identifying asymptomatic cases which may be drivers of clusters of infections (as shown in various hospital studies).

**Q21. In a public health department or infection prevention and control team, what knowledge and skills are required to interpret genomic information to support day-to-day COVID-19 cluster and outbreak risk assessment or management?**
Currently in [organisation] reliance is placed on a small number of individuals to help interpret genomic information and outside sources such as [organisation names] colleagues. Further training for a wider group of staff would be important going forward.
There is a capacity issue in IPC teams to understand WGS results and sub lineages. we need to train people to understand how to use WGS
Good working knowledge of pathogen biology, evolution and transmission, Statistics, genomics, phylogenetics and epidemiological analysis. Ideally a good working knowledge of infection control practice.

Informatics experience and at least basic knowledge of virology, epidemiology and molecular biology
Data wrangling is essential for day-to-day COVID-19 outbreak risk assessment. The ability to interpret the biological and health significance of different mutations are important in evaluating the risk of an outbreak, as well as the ability to communicate this to the lay person.
Understanding of how infections transmit and factors associated with transmission Knowledge of outbreak investigation Understanding of what sequencing data represents and how it is generated Understanding of epidemiology and probability Access to specialist expertise in microbiology/sequencing
I think an ability to understand the interpretation of lineages, sequences, SNP addresses, phylogenetic trees and the differences between them is vital. Also important is an understanding of the process for choosing/prioritizing samples and the % of samples being sequenced at a given time in order to understand context.
Relatedness of specimens. CLoseness of fit.
Need to understand the methods and the limitations of the tests done. There is little known in depts at present
If no bioinformatics skills are required, then the skills will be in the form of interpreting results. In this circumstance the following are useful: Ability to read a phylogenetic tree Understand the process and limitations of sequencing Understand the limitations of SARS-CoV-2 sequencing (low genetic diversity) The ability to combine genomic cluster data with epidemiological data (ideally at scale) Understand the IPC implications for genomically linked vs genomically refuted clusters Understand commonly used terms in genomics (SNP-distance, TMRCA, cluster, lineage, etc) Understand the limitations of epidemiological information and where genomics can help Understand the benefits of genomics in examining vaccine efficacy and reinfections Be well placed to understand the benefits of genomic sequencing for diagnostics (such as primer design).
Bioinformaticians, Bioinformaticians, Bioinformaticians!

How Public Health Authorities can use Pathogen Genomics in Health Protection Practice: A Consensus-building Delphi Study conducted in the United Kingdom

Round Two

Q1. How are you currently involved in SARS-CoV-2 genomics information flows?

Answer Choices	Responses	
None of the above	0.00%	0
Producer	8.33%	1
Consumer	75.00%	9
Both	33.33%	4
Other (please specify)		0
	**Answered**	**12**
	**Skipped**	**0**

Q2. Public health authorities should use pathogen genomic sequencing to detect the emergence of new variants.

Answer Choices	Responses	
Strongly agree	100.00%	12
Agree	0.00%	0
Neither agree nor disagree	0.00%	0
Disagree	0.00%	0
Strongly disagree	0.00%	0
	**Answered**	**12**
	**Skipped**	**0**

Q3. Public health authorities should use pathogen genomic sequencing as a surveillance tool to monitor geographical spread of variants over time.

Answer Choices	Responses	
Strongly agree	75.00%	9
Agree	25.00%	3
Neither agree nor disagree	0.00%	0
Disagree	0.00%	0
Strongly disagree	0.00%	0
	**Answered**	**12**
	**Skipped**	**0**

Q4. Public health authorities and healthcare providers should use pathogen genomic sequencing in outbreak investigations in health care settings to rule in or rule out transmission events.

Answer Choices	Responses	
Strongly agree	75.00%	9
Agree	8.33%	1
Neither agree nor disagree	16.67%	2
Disagree	0.00%	0
Strongly disagree	0.00%	0
	**Answered**	**12**
	**Skipped**	**0**

Q5. Public health authorities should use pathogen genomic data to inform estimates of epidemic growth.

Answer Choices	Responses	
Strongly agree	50.00%	6
Agree	33.33%	4
Neither agree nor disagree	16.67%	2
Disagree	0.00%	0
Strongly disagree	0.00%	0
	**Answered**	**12**
	**Skipped**	**0**

Q6. Public health authorities should use pathogen genomic data to inform estimates of clinical severity of the disease

Answer Choices	Responses	
Strongly agree	33.33%	4
Agree	58.33%	7
Neither agree nor disagree	8.33%	1
Disagree	0.00%	0
Strongly disagree	0.00%	0
	**Answered**	**12**
	**Skipped**	**0**

Q7. Public health authorities should use pathogen genomic data to detect changes in risk of infection in specific settings e.g. schools, care-homes etc.

Answer Choices	Responses	
Strongly agree	33.33%	4
Agree	41.67%	5
Neither agree nor disagree	25.00%	3
Disagree	0.00%	0
Strongly disagree	0.00%	0
	**Answered**	**12**
	**Skipped**	**0**

Q8. Pathogen genomic information should inform evaluation of the effect of:

	Strongly agree		Agree		Neither agree nor disagree		Disagree		Strongly disagree		Total	Weighted Average
non-pharmaceutical interventions	16.67%	2	33.33%	4	33.33%	4	16.67%	2	0.00 %	0	12	3.5
vaccines	66.67%	8	25.00%	3	8.33%	1	0.00%	0	0.00 %	0	12	4.58
pharmaceutical therapeutic treatments	33.33%	4	50.00%	6	16.67%	2	0.00%	0	0.00 %	0	12	4.17
											**Answered**	**12**
											**Skipped**	**0**

Q9. Pathogen genomic information should be linked to contextual epidemiological information to facilitate risk assessment.

Answer Choices	Responses	
Strongly agree	83.33%	10
Agree	8.33%	1
Neither agree nor disagree	8.33%	1
Disagree	0.00%	0
Strongly disagree	0.00%	0
	**Answered**	**12**
	**Skipped**	**0**

Q10. Public health authorities should prioritise sequencing from outbreaks where it appears there is greater than expected disease severity.

Answer Choices	Responses	
Strongly agree	58.33%	7
Agree	33.33%	4
Neither agree nor disagree	8.33%	1
Disagree	0.00%	0
Strongly disagree	0.00%	0
	**Answered**	**12**
	**Skipped**	**0**

Q11. Public health authorities should sequence enough randomly selected samples to enable unbiased surveillance

Answer Choices	Responses	
Strongly agree	75.00%	9
Agree	25.00%	3
Neither agree nor disagree	0.00%	0
Disagree	0.00%	0
Strongly disagree	0.00%	0
	**Answered**	**12**
	**Skipped**	**0**

Q12. Public health authorities should prioritise sequencing of samples from vaccinated people.

Answer Choices	Responses	
Strongly agree	25.00%	3
Agree	41.67%	5
Neither agree nor disagree	25.00%	3
Disagree	8.33%	1
Strongly disagree	0.00%	0
	**Answered**	**12**
	**Skipped**	**0**

Q13. Public health authorities should prioritise sequencing of samples from people who have had multiple episodes of infection.

Answer Choices	Responses	
Strongly agree	33.33%	4
Agree	50.00%	6

Neither agree nor disagree	8.33%	1
Disagree	8.33%	1
Strongly disagree	0.00%	0
	**Answered**	**12**
	**Skipped**	**0**

Q14. If it is not possible to sequence all samples, public health authorities should direct sequencing capacity towards vulnerable populations.

Answer Choices	Responses	
Strongly agree	8.33%	1
Agree	58.33%	7
Neither agree nor disagree	8.33%	1
Disagree	25.00%	3
Strongly disagree	0.00%	0
	**Answered**	**12**
	**Skipped**	**0**

Q15. If it is not possible to sequence all samples, public health authorities should direct sequencing capacity towards outbreak investigation.

Answer Choices	Responses	
Strongly agree	25.00%	3
Agree	33.33%	4
Neither agree nor disagree	25.00%	3
Disagree	16.67%	2
Strongly disagree	0.00%	0
	**Answered**	**12**
	**Skipped**	**0**

Q16. If it is not possible to sequence all samples, public health authorities should direct sequencing capacity towards populations in whom new variants might be present e.g. international travellers and immunocompromised people.

Answer Choices	Responses	
Strongly agree	58.33%	7
Agree	41.67%	5
Neither agree nor disagree	0.00%	0
Disagree	0.00%	0
Strongly disagree	0.00%	0
	**Answered**	**12**
	**Skipped**	**0**

Q17. Public health authorities should use pathogen genomic data to de-escalate potential outbreaks that were identified through epidemiological links.

Answer Choices	Responses	
Strongly agree	8.33%	1
Agree	50.00%	6
Neither agree nor disagree	33.33%	4
Disagree	8.33%	1
Strongly disagree	0.00%	0
	**Answered**	**12**
	**Skipped**	**0**

Q18. Timeliness of genomic sequencing results is important to allow results to be acted upon by public health authorities.

Answer Choices	Responses	
Strongly agree	75.00%	9
Agree	25.00%	3
Neither agree nor disagree	0.00%	0
Disagree	0.00%	0
Strongly disagree	0.00%	0
	**Answered**	**12**
	**Skipped**	**0**

Q19. Clinical teams need timely access to sequence results to inform treatment and infection control decisions.

Answer Choices	Responses	
Strongly agree	58.33%	7
Agree	33.33%	4
Neither agree nor disagree	0.00%	0
Disagree	8.33%	1
Strongly disagree	0.00%	0
	**Answered**	**12**
	**Skipped**	**0**

Q20. A minimum of ten percent of all positive covid-19 samples should be sequenced.

Answer Choices	Responses	
Strongly agree	33.33%	4
Agree	25.00%	3
Neither agree nor disagree	41.67%	5

Disagree	0.00%	0
Strongly disagree	0.00%	0
	**Answered**	**12**
	**Skipped**	**0**

Q21. Public health authorities should use tools such as rapid genotyping for surveillance of lineages.

Answer Choices	Responses	
Strongly agree	33.33%	4
Agree	41.67%	5
Neither agree nor disagree	25.00%	3
Disagree	0.00%	0
Strongly disagree	0.00%	0
	**Answered**	**12**
	**Skipped**	**0**

Q22. Public health authorities should analyse sequence and/or single nucleotide polymorphism data as part of investigation of outbreaks.

Answer Choices	Responses	
Strongly agree	25.00%	3
Agree	41.67%	5
Neither agree nor disagree	25.00%	3
Disagree	8.33%	1
Strongly disagree	0.00%	0
	**Answered**	**12**
	**Skipped**	**0**

Q23. Analysts should exclude sequences below a defined sequence coverage from analysis when investigating transmission events.

Answer Choices	Responses	
Strongly agree	16.67%	2
Agree	16.67%	2
Neither agree nor disagree	50.00%	6
Disagree	8.33%	1
Strongly disagree	8.33%	1
	**Answered**	**12**
	**Skipped**	**0**

Q24. Public health authorities should use bioinformatics tools to elicit unsuspected transmission events.

Answer Choices	Responses	
Strongly agree	25.00%	3
Agree	58.33%	7
Neither agree nor disagree	16.67%	2
Disagree	0.00%	0
Strongly disagree	0.00%	0
	**Answered**	**12**
	**Skipped**	**0**

Q25. Public health authorities should ensure training is provided for health protection, infection control and clinical teams on the interpretation of sequencing results.

Answer Choices	Responses	
Strongly agree	100.00%	12
Agree	0.00%	0
Neither agree nor disagree	0.00%	0
Disagree	0.00%	0
Strongly disagree	0.00%	0
	**Answered**	**12**
	**Skipped**	**0**

Q26. Additional comments

**Answered**	**2**
**Skipped**	**10**

Round Three

Q1. How are you currently involved in SARS-CoV-2 genomics information flows?

Answer Choices	Responses	
None of the above	0.00%	0
Producer	7.14%	1
Consumer	64.29%	9
Both	28.57%	4
Other (please specify)		0
	**Answered**	**14**
	**Skipped**	**0**

Q2. If it is not possible to sequence all samples, public health authorities should direct sequencing capacity towards people who are at greater risk of adverse outcomes from infection.

Answer Choices	Responses	
Strongly agree	28.57%	4
Agree	42.86%	6
Neither agree nor disagree	0.00%	0
Disagree	28.57%	4
Strongly disagree	0.00%	0
	**Answered**	**14**
	**Skipped**	**0**

Q3. If it is not possible to sequence all samples, public health authorities should direct sequencing capacity towards outbreak investigations:

	Strongly agree		Agree		Neither agree nor disagree		Disagree		Strongly disagree		Total	Weighted Average
In closed settings, such as care homes and hospitals.	64.29%	9	28.57%	4	7.14%	1	0.00%	0	0.00%	0	14	1.43
In community outbreaks, such as at public events or functions.	21.43%	3	50.00%	7	28.57%	4	0.00%	0	0.00%	0	14	2.07
											**Answered**	**14**
											**Skipped**	**0**

Q4. Public health authorities should use pathogen genomic data to de-escalate potential outbreaks that were identified through epidemiological links:

	Strongly agree		Agree		Neither agree nor disagree		Disagree		Strongly disagree		Total	Weighted Average
In closed settings, such as care homes and hospitals.	35.71%	5	57.14%	8	7.14%	1	0.00%	0	0.00%	0	14	1.71
In community outbreaks, such as at public events or functions.	14.29%	2	57.14%	8	21.43%	3	7.14%	1	0.00%	0	14	2.21
											**Answered**	**14**
											**Skipped**	**0**

Q5. Pathogen genomic information should inform evaluation of the effect of non-pharmaceutical interventions.

Answer Choices	Responses	
Strongly agree	14.29%	2
Agree	57.14%	8
Neither agree nor disagree	28.57%	4
Disagree	0.00%	0
Strongly disagree	0.00%	0
	**Answered**	**14**
	**Skipped**	**0**

Q6. The proportion of samples sequenced should reflect the epidemiological context.

Answer Choices	Responses	
Strongly agree	35.71%	5
Agree	50.00%	7
Neither agree nor disagree	14.29%	2
Disagree	0.00%	0
Strongly disagree	0.00%	0
	**Answered**	**14**
	**Skipped**	**0**

Q7. Public health authorities should analyse sequencing data in more detail than lineage as part of the investigation of outbreaks.

Answer Choices	Responses	
Strongly agree	42.86%	6
Agree	50.00%	7
Neither agree nor disagree	0.00%	0
Disagree	7.14%	1
Strongly disagree	0.00%	0
	**Answered**	**14**
	**Skipped**	**0**

Q8. Analysts should only include sequences above defined sequence coverage depth when investigating transmission events. (This is an overall quality control value provided for all sequencing results).

Answer Choices	Responses	
Strongly agree	7.14%	1
Agree	50.00%	7
Neither agree nor disagree	28.57%	4
Disagree	7.14%	1
Strongly disagree	7.14%	1

**Answered**	**14**
**Skipped**	**0**

Q9. Additional comments

**Answered**	**3**
**Skipped**	**11**
